# Amorphous Calcium Carbonate Granules Form Within an Intracellular Compartment in Calcifying Cyanobacteria

**DOI:** 10.3389/fmicb.2018.01768

**Published:** 2018-08-06

**Authors:** Marine Blondeau, Martin Sachse, Claire Boulogne, Cynthia Gillet, Jean-Michel Guigner, Fériel Skouri-Panet, Mélanie Poinsot, Céline Ferard, Jennyfer Miot, Karim Benzerara

**Affiliations:** ^1^UMR CNRS 7590, IRD, Muséum National d’Histoire Naturelle, Institut de Minéralogie, de Physique des Matériaux et de Cosmochimie, Sorbonne Université, Paris, France; ^2^Unité Technologie et Service BioImagerie Ultrastructurale, Citech, Institut Pasteur, Paris, France; ^3^CEA, Centre National de la Recherche Scientifique, Institute for Integrative Biology of the Cell (I2BC), Université Paris-Sud, Université Paris-Saclay, Gif-sur-Yvette, France

**Keywords:** amorphous calcium carbonate, cyanobacteria, biomineralization, freeze-substitution, CEMOVIS, bacterial microcompartment, carboxysome, thylakoid

## Abstract

The recent discovery of cyanobacteria forming intracellular amorphous calcium carbonate (ACC) has challenged the former paradigm suggesting that cyanobacteria-mediated carbonatogenesis was exclusively extracellular. Yet, the mechanisms of intracellular biomineralization in cyanobacteria and in particular whether this takes place within an intracellular microcompartment, remain poorly understood. Here, we analyzed six cyanobacterial strains forming intracellular ACC by transmission electron microscopy. We tested two different approaches to preserve as well as possible the intracellular ACC inclusions: (i) freeze-substitution followed by epoxy embedding and room-temperature ultramicrotomy and (ii) high-pressure freezing followed by cryo-ultramicrotomy, usually referred to as cryo-electron microscopy of vitreous sections (CEMOVIS). We observed that the first method preserved ACC well in 500-nm-thick sections but not in 70-nm-thick sections. However, cell ultrastructures were difficult to clearly observe in the 500-nm-thick sections. In contrast, CEMOVIS provided a high preservation quality of bacterial ultrastructures, including the intracellular ACC inclusions in 50-nm-thick sections. ACC inclusions displayed different textures, suggesting varying brittleness, possibly resulting from different hydration levels. Moreover, an electron dense envelope of ∼2.5 nm was systematically observed around ACC granules in all studied cyanobacterial strains. This envelope may be composed of a protein shell or a lipid monolayer, but not a lipid bilayer as usually observed in other bacteria forming intracellular minerals. Overall, this study evidenced that ACC inclusions formed and were stabilized within a previously unidentified bacterial microcompartment in some species of cyanobacteria.

## Introduction

The cyanobacterium *Gloeomargarita lithophora*, isolated from microbialites in the alkaline Lake Alchichica (Mexico), was reported to form intracellular amorphous Ca-Mg-Sr-Ba-carbonates (Couradeau et al., 2012; Moreira et al., 2017). This discovery was particularly surprising since cyanobacterium-mediated carbonatogenesis was before then thought to be exclusively extracellular (e.g., Riding, 2006; Dittrich and Sibler, 2010). Moreover, intracellular biomineralization of carbonates by *G. lithophora* was shown to be associated with the capability to strongly accumulate Sr and Ba offering some potential for remediating pollutions by these elements (Cam et al., 2016; Blondeau et al., 2018). Finally, *G. lithophora* was recently proposed as the present-day closest cultured relative of primary plastids (Ponce-Toledo et al., 2017), questioning the capability of their common ancestor to form intracellular CaCO_3_.

A broad diversity of cyanobacterial species from diverse environments has been found to also form intracellular amorphous calcium carbonates (ACC), showing that this biomineralization mechanism was widespread geographically and phylogenetically (Benzerara et al., 2014; Ragon et al., 2014). At least two patterns of carbonate spatial distribution within the cells were described (Benzerara et al., 2014; Li et al., 2016): (i) in a first group of phylogenetically diverse strains, ACC granules were scattered or aligned within cells. (ii) In a second group corresponding to one single clade including the strain *Thermosynechococcus elongatus* BP-1, ACC granules were shown to nucleate at the septum during cell division and were eventually located at the cell poles.

However, the cellular and molecular mechanisms involved in the intracellular biomineralization of ACC within cyanobacteria remain poorly understood. Cyanobacteria are known to regulate their cytoplasmic pH at values of ∼6.8–7.9 (Belkin and Boussiba, 1991; Jiang et al., 2013), the cytoplasmic concentration of HCO_3_^−^ at ∼30 mM (Badger and Andrews, 1982; Skleryk et al., 1997) and the cytoplasmic concentration of Ca^2+^ at ∼100–200 nM (Torrecilla et al., 2000; Barrán-Berdón et al., 2011). However, these conditions are thermodynamically inconsistent with the precipitation of ACC because such an environment is undersaturated with all Ca-carbonate phases (Cam et al., 2015). Several hypotheses have been proposed to address this paradox: (i) one or several of these chemical parameters may be very different in the cytoplasm of cyanobacteria forming intracellular carbonates (Cam et al., 2015); (ii) ACC form within intracellular vesicles, where [Ca^2+^], [HCO_3_^−^] and/or pH may be much higher than in the cytoplasm (Cam et al., 2018); (iii) ACC may form within carboxysomes, i.e., compartments where CO_2_ is fixed by RuBisCO within cyanobacterial cells (Li et al., 2016). One way to challenge these different hypotheses would consist in imaging cell ultrastructures associated with intracellular carbonates. For example, this has been achieved for *Achromatium oxaliferum*, a member of Gammaproteobacteria, forming intracellular calcite, i.e., a crystalline form of Ca-carbonates (Gray and Head, 2014). In this case, transmission electron microscopy (TEM) analyses of cells prepared by conventional ultramicrotomy, suggested that calcite precipitation occurred within vacuoles/vesicles. De Boer et al. (1971) mentioned that these vacuoles were enclosed by a single-layer membrane, whereas Head et al. (2000) and Gray and Head (2014) recognized a lipid bilayer. However, this approach was unsuccessful when applied to cyanobacteria forming intracellular ACC. Indeed, chemical fixatives and organic solvents used by conventional ultramicrotomy induced complete dissolution of intracellular ACC, which is more soluble than calcite (Li et al., 2016). Several methods of biological sample preparation alternative to conventional ultramicrotomy have been previously developed (e.g., Edelmann, 2002; Cavalier et al., 2009; Frankl et al., 2015). For example, freeze-substitution has been shown to preserve cell ultrastructures very well, including membranes, the cytoplasm and DNA (e.g., Giddings, 2003; Bleck et al., 2010). In this preparation process, the sample is frozen as amorphous ice (vitrified), dehydrated and chemically fixed at low temperatures (below 0°C) before embedding in resin at room temperature (Feder and Sidman, 1958; Humbel, 2009). Alternatively, a technique called cryo-electron microscopy of vitreous sections (CEMOVIS), allows the observation of cell ultrastructures preserved near their native state in amorphous ice (Adrian et al., 1984; Matias et al., 2003; Al-Amoudi et al., 2004; Dubochet, 2012). In this approach, the sample is first vitrified, then sectioned and observed in the vitreous state by cryo-electron microscopy (Dubochet et al., 2009). On the contrary to other techniques, CEMOVIS does not involve the use of chemical fixatives and staining agents to preserve and contrast the cell structures (Eltsov and Dubochet, 2005).

Here, we studied the ultrastructures of several cyanobacterial strains forming intracellular ACC with different spatial distribution patterns. Two approaches were used to determine whether ACC were enclosed within an intracellular compartment: (1) freeze-substitution followed by resin embedding and (2) CEMOVIS. The picture provided by these methods on the ultrastructures of cyanobacteria forming intracellular ACC was more accurate than that given by conventional ultramicrotomy and offered the possibility to test existing hypotheses on the mechanisms of intracellular ACC biomineralization.

## Materials and Methods

### Cyanobacterial Strains and Culture Conditions

Six cyanobacterial strains forming intracellular ACC were studied. *G. lithophora* C7 and *Synechococcus calcipolaris* G9 were isolated from Lake Alchichica. *G lithophora* was described by Moreira et al. (2017). Three axenic cyanobacteria were obtained from the Pasteur Collection of Cyanobacteria: *Cyanothece* sp. PCC 7425 was isolated from a rice paddy in Senegal (Rippka et al., 1979; Porta et al., 2000); *Synechococcus* sp. PCC 6312 was isolated from a freshwater pond in California (Stanier et al., 1971; Rippka et al., 1979); *Synechococcus* sp. PCC 6717 was isolated from a hot spring in Yellowstone (United States) (Stanier et al., 1971; Rippka et al., 1979). *T. elongatus* BP-1, a thermophilic cyanobacterium was isolated from a hot spring in Beppu (Japan) (Yamaoka et al., 1978). These strains were cultured in the BG-11 medium under continuous light at 30°C, except for *T. elongatus* which was grown at 45°C. The chemical composition of the BG-11 medium was 17.65 mM NaNO_3_, 0.18 mM K_2_HPO_4_, 0.3 mM MgSO_4_, 0.25 mM CaCl_2_, 0.03 mM citric acid, 0.03 mM ferric ammonium citrate, 0.003 mM ethylenediaminetetraacetic acid (EDTA), 0.38 mM Na_2_CO_3_, and trace minerals (Rippka et al., 1979). Cells were sampled in the exponential phase and prepared for electron microscopy (Supplementary Table S1).

### Sample Preparation for (Cryo)-Electron Microscopy

#### High-Pressure-Freezing and Freeze-Substitution

Between 1 and 2 mL of the cultures were centrifuged at 5000 *g* for 10 min. Cell pellets were placed in copper carriers filled with hexadecene. Samples were then vitrified using a high-pressure freezer EMPACT2 (Leica). Vitrified samples, preserved in liquid nitrogen, were thereafter placed in freeze-substitution cocktail flasks at −90°C in an Automate Freeze-Substitution (AFS2). The substitution medium contained 2% OsO_4_ and 2.5% H_2_O in acetone. The freeze-substitution was performed according to the following procedure: −90°C for 20 h; warming up to −60°C at 2°C per hour; −60°C for 8 h; warming up to −30°C at 2°C per hour; −30°C for 8 h; washing in pure acetone (three times every 10 min). Finally, samples were progressively embedded (i) in a 1/3 epoxy/aceton mixture for 2 h at −30°C before warming up to 0°C (15°C per hour); (ii) in a 1/1 epoxy/acetone mixture for 2 h at room temperature; (iii) in a 3/1 epoxy/acetone mixture overnight at room temperature.

Samples were then incubated three times in pure epoxy for 2 h and finally, in a new bath of pure epoxy overnight before polymerization at 60°C for 18–24 h. Ultrathin (70 nm) and semithin (250–500 nm) sections were cut using a diamond knife (45° angle) on a Leica UC6 ultramicrotome. Sections were collected on Formvar^TM^ carbon-coated copper grids. Some sections were stained with uranyl acetate at 2% for 15 min, washed three times with milliQ water and dried at room temperature.

#### CEMOVIS

For CEMOVIS, between 25 and 50 mL of the cultures were centrifuged at 4400 *g* during 10 min. The processed culture volumes were determined so that the final cell density before vitrification was ∼6.10^9^ cells/mL. Supernatants were discarded and pellets were suspended in 200 μL of the culture medium. Then, 200 μL of dextran at 40% in BG-11 were added. The suspension was then transferred in gold tubes and vitrified using a Baltec HPM010 high pressure freezer (Abra-fluid, Widnau, Switzerland). Vitrified samples were placed in the cryo-chamber of a Leica cryo-ultramicrotome at −140°C. Gold tubes containing vitrified samples were mounted on aluminum pins using a cryo-glue, which consisted in a mixture of ethanol: 2-propanol (=2:3 v/v) (Chlanda and Sachse, 2014). The cryo-chamber was cooled down to −160°C. At this temperature, the glue was hard enough to withstand the sectioning stress. Ultrathin cryo-sections measuring ∼50 nm in thickness were obtained using a 35° cryo-diamond knife and were collected on quantifoil grids covered with a carbon film. Grids were stored in liquid nitrogen before analysis.

#### Air Dried Cells

Non-sectioned whole cells were also observed by scanning transmission electron microscopy (STEM). These samples were prepared by centrifuging cultures at 5000 *g* during 10 min. Pellets were washed three times in Milli-Q water. Two microliters of the final suspension were deposited on Formvar^TM^ carbon-coated copper grid and dried at room temperature.

### Transmission Electron Microscopy (TEM) Analyses

Air-dried cells and sections prepared by freeze substitution were observed at room temperature using a TEM JEOL 2100F, equipped with field emission gun and operating at 200 kV. STEM images were acquired in the high angle annular dark field (HAADF) mode. Energy dispersive x-ray spectrometry (EDXS) analyses were processed using the JEOL Analysis Station software.

CEMOVIS sections were observed using a TEM FEI Tecnai F20 operating at 200 kV in the low dose mode to reduce beam damages. Images were recorded with a series of varying defocus. EDXS spectra were acquired on CEMOVIS sections using a TEM JEOL 2100, equipped with a LaB6 filament, operating at 80 or 200 kV.

## Results

### Analyses of Ultramicrotomy Sections Prepared by Freeze-Substitution

**FIGURE 1:**
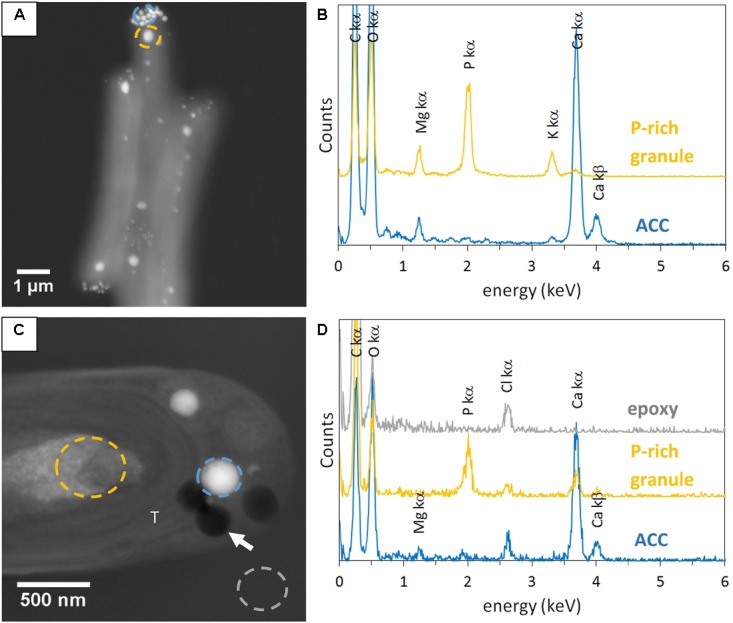
STEM-HAADF and EDXS analyses of *Synechococcus* sp. PCC 6717. **(A)** Image of air-dried un-sectioned cells. **(B)** EDXS spectra of an ACC inclusion and a P-rich granule. See **A** for locations. **(C)** Image of a cell in a 500-nm-thick unstained section. **(D)** EDXS spectra of an ACC inclusion, a P-rich granule, and epoxy. See **C** for location. The white arrow shows one of the holes in the section. T, thylakoid.

**FIGURE 2:**
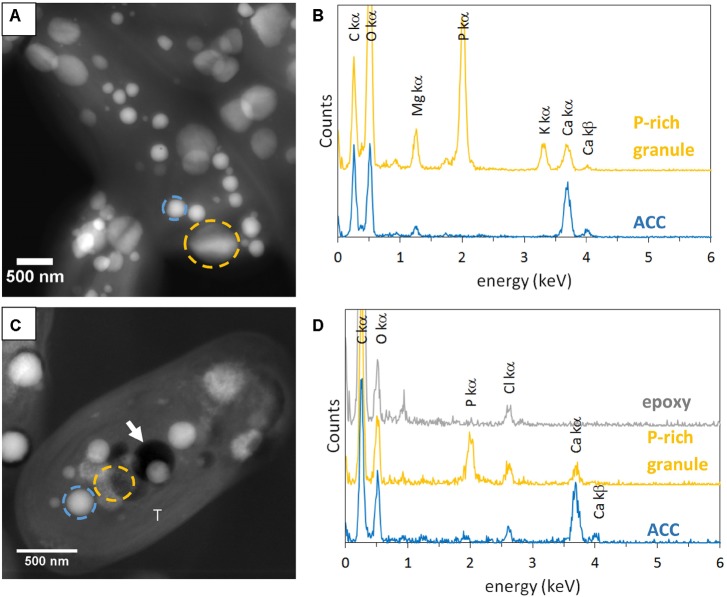
STEM-HAADF and EDXS analyses of *Gloeomargarita lithophora*. **(A)** STEM-HAADF image of air-dried un-sectioned cells. **(B)** EDXS spectra of an ACC inclusion and a P-rich granule. See **A** for locations. **(C)** STEM-HAADF image of a cell in a 500-nm-thick unstained sections. **(D)** EDXS spectra extracted of an ACC inclusion, a P-rich granule and epoxy. See **C** for locations. The white arrow shows a hole in resin. T, thylakoid.

Some electron dense ACC inclusions were well-preserved in 500-nm-thick unstained sections, as shown by the detection of Ca-rich and P-free granules in *Synechococcus* sp. PCC 6717 cells (**Figure 1**). As expected, these inclusions were located preferentially at the cell poles in *Synechococcus* sp. PCC 6717, PCC6312, *CandidatusSynechococcus calcipolaris* G9 and *T. elongatus* BP-1. This spatial distribution was similar to that of ACC inclusions in air-dried un-sectioned cells. Moreover, sections showed that ACC inclusions in these strains were located between the cytoplasmic membrane and thylakoids (**Figure 1** and Supplementary Figures S1–S3). In contrast, ACC inclusions were located centrally within the cells of *G. lithophora* C7 and *Cyanothece* sp. PCC 7425 and more internally to the cell compared to the peripheral thylakoids (**Figure 2** and Supplementary Figure S4). The presence of circular holes in the sections with sizes similar to those of ACC inclusions suggested that some inclusions were lost during the sectioning process (**Figures 1**, **2** and Supplementary Figures S1–S4). In 500-nm-thick unstained sections, fibrous coarse non-spherical granules were also observed in the cytoplasm of cells of all strains (**Figures 1**, **2** and Supplementary Figures S1–S4). They appeared darker than ACC inclusions by STEM-HAADF and were located more internally to the cells compared to the thylakoids, sometimes adjacent to the ACC inclusions such as in *G. lithophora* (**Figure 2**). According to EDXS analyses, these granules were mostly composed of P with some Mg and K in un-sectioned whole cells, while they contained P with some Ca but no Mg and K in sections (**Figures 1**, **2** and Supplementary Figures S1–S4).

**FIGURE 3:**
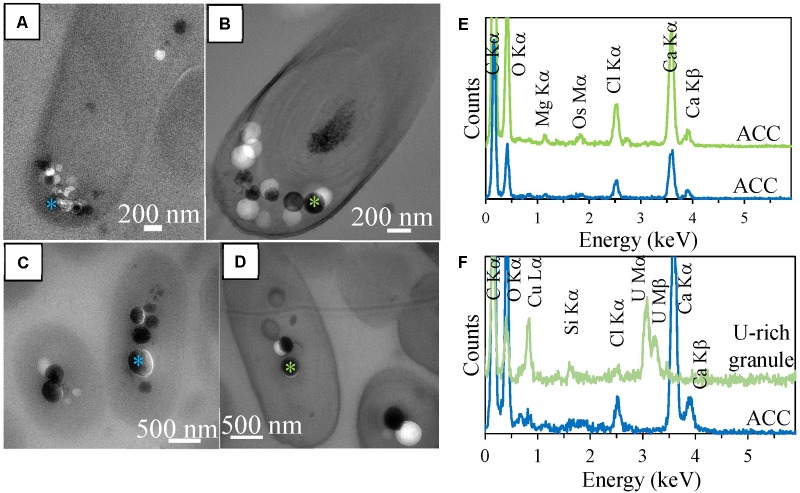
TEM analyses of 500-nm-thick sections of *Synechococcus* sp. PCC 6717 and *Cyanothece* sp. PCC 7425. **(A)** Unstained section of a *Synechococcus* sp. PCC 6717 cell. **(B)** Stained section of a *Synechococcus* sp. PCC 6717 cell. **(C)** EDXS spectra of ACC inclusions from the stained (upper spectrum) and the unstained (lower spectrum) sections. **(D)** Unstained section of a *Cyanothece* sp. PCC 7425 cell. **(E)** Stained section of a *Cyanothece* sp. PCC 7425 cell. **(F)** EDXS spectra of an ACC inclusion in the unstained section (lower spectrum) and a U-rich granule in the stained section. Chloride and silicon are contained in epoxy resin.

In order to better contrast cell ultrastructures, some sections were stained by uranyl acetate. In 500-nm-thick stained sections, several ACC inclusions were well-preserved in *Synechococcus sp.* PCC 6717 cells (**Figure 3**). EDXS analyses showed that they were composed of Ca mostly, with some Mg. However, in stained sections of *Cyanothece sp.* PCC 7425, some inclusions which appeared similar to ACC inclusions based on their size and localization were mostly composed of U, suggesting dissolution of ACC upon staining and precipitation of a U-rich phase pseudomorphizing the original ACC granules (**Figures 3E,F**).

**FIGURE 4:**
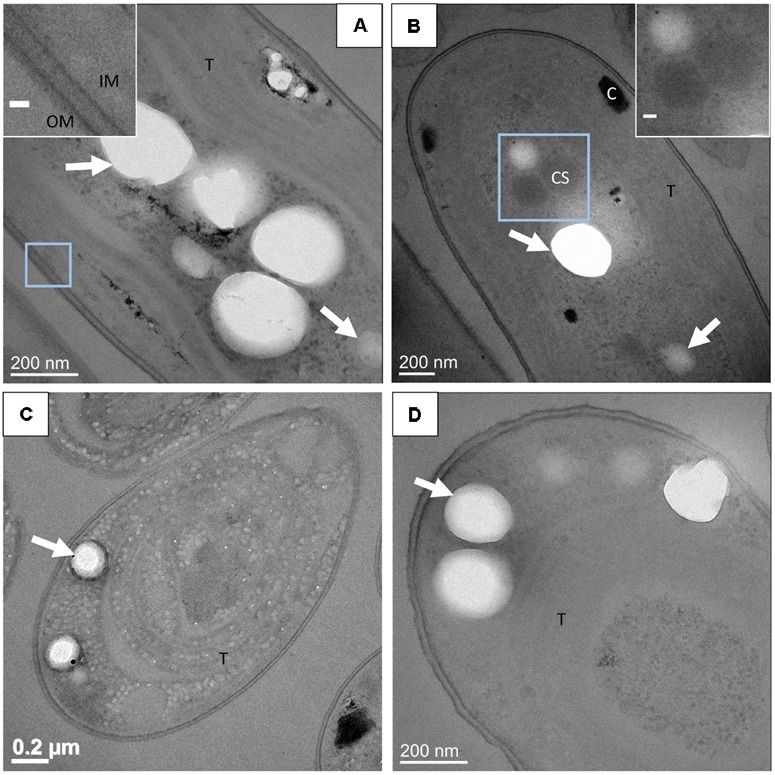
TEM images of stained 70-nm-thick sections. **(A)** *Gloeomargarita lithophora*. Scale bar in the insert: 20 nm. **(B)** *Cyanothece* sp. PCC 7425. Scale bar in the insert: 50 nm. **(C)** *Synechococcus* sp. PCC 6312. **(D)** *Synechococcus* sp. PCC 6717. T, thylakoid; CS, carboxysome; C, cyanophycin granule; IM, inner membrane; OM, outer membrane. White arrows show holes in resin and electron-transparent bodies.

In stained and unstained 500-nm-thick sections, details of the cell ultrastructures were difficult to image because of the relatively high thickness of the sections. Thinner sections of ∼70 nm in thickness were therefore cut and observed. The cell ultrastructures of *G. lithophora*, *Cyanothece* sp., *Synechococcus* sp. PCC 6312 and 6717 were visible in 70-nm-thick stained sections prepared by freeze-substitution (**Figure 4**). Cell walls, comprising the inner and the outer membranes, were clearly observed. Thylakoids were visible, although their membranes were not clearly distinguished. Slightly contrasted intracellular compartments with a polyhedral shape, interpreted as carboxysomes were detected repeatedly (**Figure 4B**). High electron-dense bodies, located between thylakoids and the cytoplasmic membrane were observed in *Cyanothece sp.* PCC 7425 (**Figure 4B**). They might correspond to cyanophycin granules in accordance with the study by Porta et al. (2000). No electron-dense ACC inclusion was detected in stained and unstained 70-nm-thick sections (Supplementary Figure S5). However, nearly circular electron-transparent holes were observed in stained and unstained 70-nm-thick sections for all the strains. They had a size, morphology and spatial distribution similar to the ACC inclusions detected in unstained 500-nm-thick sections (**Figures 1**, **2**, **4** and Supplementary Figures S3,S4). Therefore, this suggested that ACC inclusions were lost during the cutting of 70-nm-thick sections.

**FIGURE 5:**
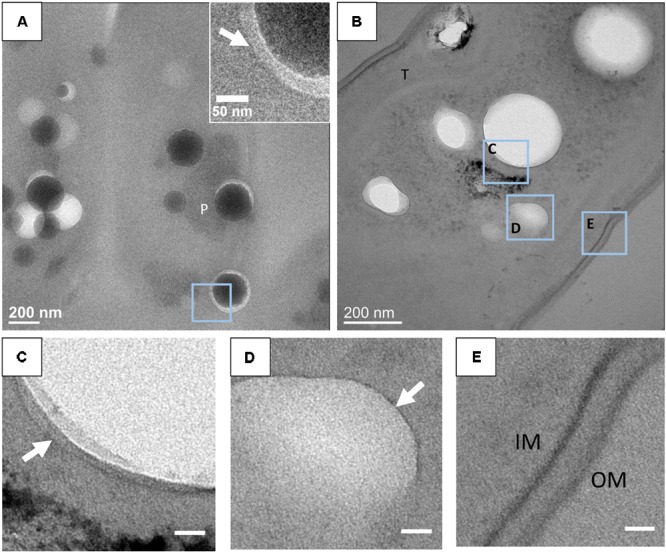
**(A)** TEM image of an unstained 500-nm-thick section of *G. lithophora*. The insert shows the edge of an ACC inclusion separated from resin by a gap. The white arrow shows a dark rim in the resin. **(B)** TEM image of a stained 70-nm-thick section of a *G. lithophora* cell. **(C)** Close-up of the area C outlined in **B**, showing a thin layer (white arrow) in the resin, around the hole. **(D)** Close-up of the area **D** outlined in **B**, showing an electron-transparent body, surrounded by a thin structure (white arrow). **(E)** Close-up of the area **E**, outlined in **B**, showing the cytoplasmic membrane (IM) and the outer membrane (OM) of the cell. P, P-rich granule; T, thylakoid. Scale bars in **C–E**: 20 nm.

In some sections, a thin electron-dense linear feature was observed around ACC inclusions (**Figure 5**). This feature could sometimes be confused with the rim of the holes created by the loss of ACC inclusions. However, at least in some occurrences, these thin features were clearly imaged within the epoxy and not at the interface between epoxy and the holes (**Figure 5C**). These layers appeared as electron-dense single lines in contrast to the two parallel electron-dense lines observed in phospholipid bilayers. They measured 1.1 +/- 0.1 nm in width. By comparison, the cytoplasmic membranes measured 5.8 +/- 0.7 nm in width (**Figures 5C–E**).

Overall, the artefactual loss of inclusions in the 70-nm-thick sections and the difficulty to image detailed ultrastructural features of the cells in 500-nm-thick sections called for the use of CEMOVIS as an alternative approach to better assess the relationships between cell ultrastructure and ACC inclusions.

### Analyses of Cells by CEMOVIS

**FIGURE 6:**
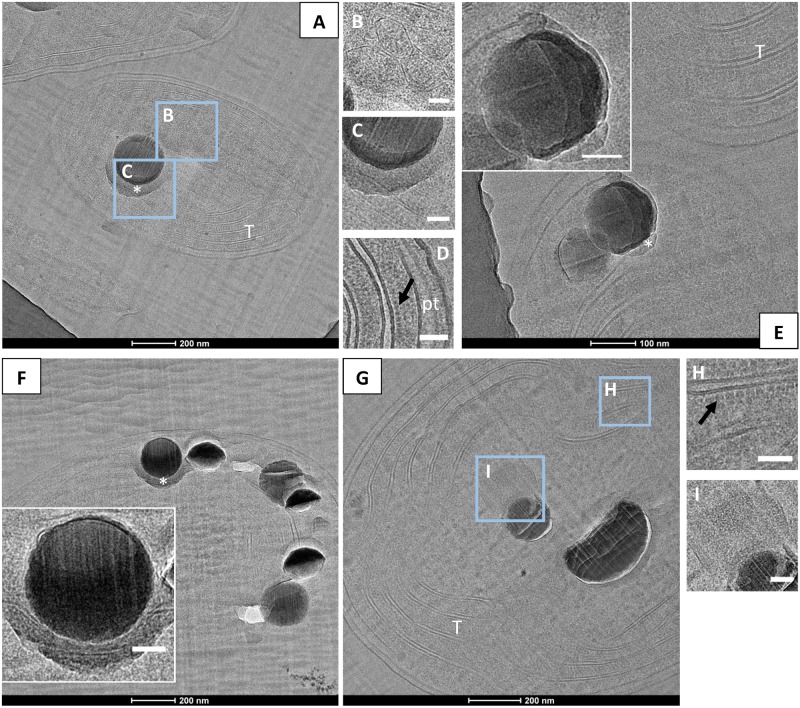
CEMOVIS analyses of cyanobacteria forming intracellular ACC. **(A–D)** Bright-field TEM images of *G. lithophora*. **(A)** The cell in the center of the micrograph contains an ACC inclusion. A medium-dark smear, marked by a white asterisk, can be observed under the inclusion. Thylakoids (T) are observed. **(B)** Close-up of carboxysomes (see location outlined in **A**). **(C)** Close-up of the ACC inclusion and the bottom border (see location outlined in **A**). **(D)** Close-up of the cell envelope showing, on the right, the inner and outer membranes as well as the peptidoglycane (pt), and two thylakoids on the left with phycobilisomes (dark arrow) at the surface and between thylakoids. **(E)** The poles of two cells of *Synechococcus* sp. PCC 6312 are visible. The bottom cell shows two ACC inclusions located between the cell envelope on the top and thylakoids (T) in the bottom. A bottom layer bordering an ACC inclusion is labeled with a white asterisk. The insert shows a close-up of one of the ACC inclusions. **(F)** One cell of *Synechococcus* sp. PCC 6717 can be observed. The insert shows a close-up of one ACC inclusion with a bottom border. **(G–I)** Bright-field TEM images of *Cyanothece* sp. PCC 7425. **(G)** The cell contains two ACC inclusions. **(H)** Close-up of the thylakoid area (see location outlined in **G**), showing aligned phycobilisomes (dark arrow) and lipid bilayers. **(I)** Close-up of a carboxysome close to the ACC inclusion (marked **I** in part **G**). Scale bars: (**B–D**,**H**,**I**, inserts in **E**,**F**) 50 nm.

CEMOVIS provided clear pictures of very well preserved cell ultrastructures. Lipid bilayers of the cell envelopes as well as the thylakoids were visible. Moreover, the peptidoglycan layer between the inner and the outer membranes was observed repeatedly (**Figure 6**). Phycobilisomes at the surface of thylakoids and protein units composing the shell of carboxysomes were also clearly detected (**Figures 6B,D,H**). The mean size of the carboxysomes varied between 130 and 270 nm depending on the cyanobacterial strains (Supplementary Table S2). Moreover, smooth electron-dense inclusions were identified as ACC inclusions based on their size, morphology, cellular localization and chemical composition as analyzed by EDXS (**Figure 6** and Supplementary Figure S6). For *G. lithophora* and *Cyanothece sp.* PCC 7425, ACC inclusions were located at the center of the cells and sometimes near thylakoids or carboxysomes, without showing any evidence of direct connections with these compartments (**Figures 6A–C,G,I**). For *Synechococcus sp.* PCC 6717 and PCC 6312, ACC inclusions were located between the cytoplasmic membrane and the outermost thylakoids (**Figures 6E,F**). ACC inclusions had a mean apparent diameter of 214 ± 68 nm (*n* = 63) for *G. lithophora*, 307 ± 89 (*n* = 30) for *Cyanothece sp.*, 163 ± 40 nm (*n* = 64) for *Synechococcus sp.* PCC 6717 and 164 ± 54 nm (*n* = 28) for *Synechococcus sp.* PCC 6312. Apparent diameters depended on the actual size of the inclusions as well as the distance at which inclusions were cut from their center. On one side of the inclusions, downstream to the cut, an electron dense area, appearing as a smear leaking from the ACC inclusions, was often observed (**Figures 6A,E,F**). Some ACC inclusions showed fractures perpendicular to the cut direction (**Figure 6G**), while other inclusions were very smooth (**Figure 6E**). Finally, several ACC inclusions were homogeneous in contrast, while others showed heterogeneities suggesting that they were not homogeneously filled spheres (**Figure 6**).

**FIGURE 7:**
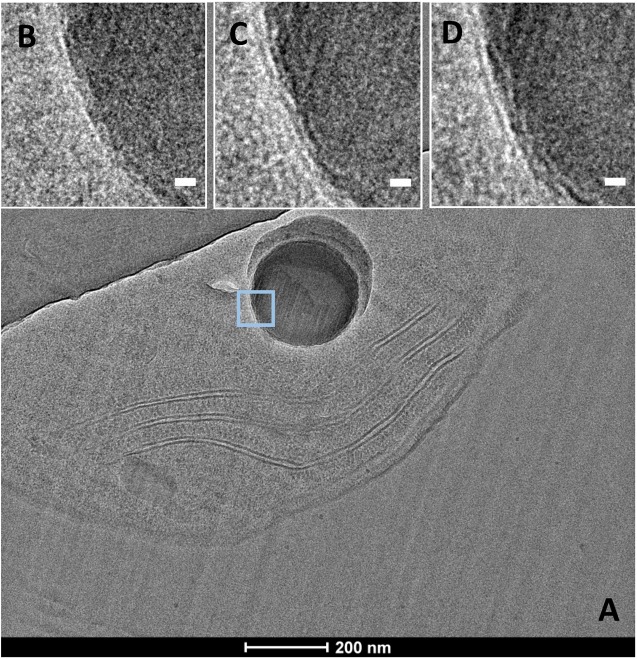
CEMOVIS images of *G. lithophora*. **(A)** Image a cell with one ACC inclusion. **(B–D)** Close-ups of the ACC inclusion (see outline in **A**), showing a membrane/shell around the inclusion (white arrows). Images were acquired at several focus heights: **(B)** –1 μm, **(C)** –2 μm, and **(D)** –3 μm. Scale bars: **(B**–**D)** 10 nm.

**FIGURE 8:**
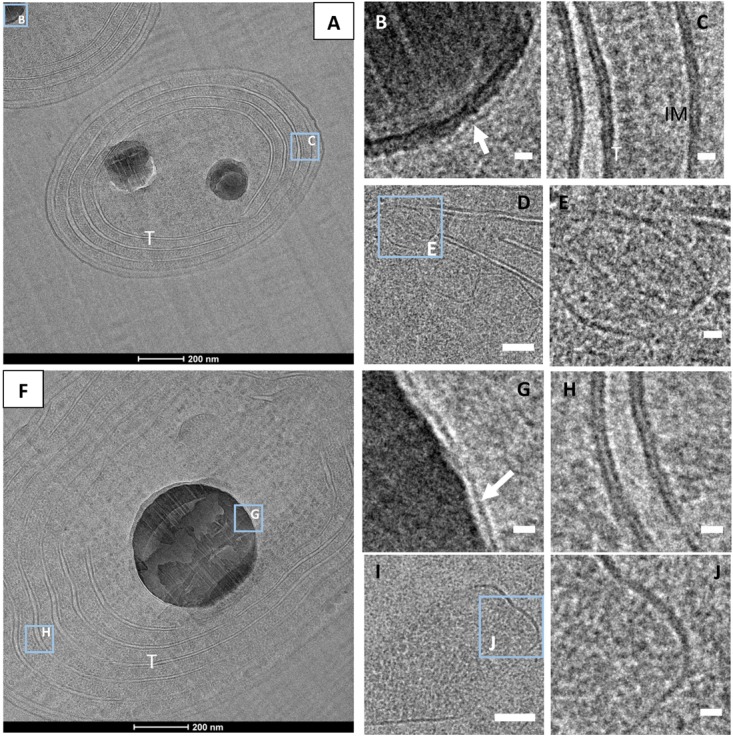
CEMOVIS images. **(A)** *G. lithophora* cells **(B)** Close-up of one ACC inclusion (see outline in **A** for location), showing a membrane/shell around the inclusion (arrow). **(C)** Close-up of an area showing the lipid bilayers of thylakoid and the cytoplasmic membranes (see outline **C** in **A**). **(D)** Carboxysomes close to thylakoids. **(E)** Close-up of the protein shell of a carboxysome (see outline in **D**). **(F)** *Cyanothece* sp. cell showing a central ACC inclusion **(G)** Close-up of the ACC inclusion (see outline in **F**), showing a membrane/shell around. **(H)** Close-up of a thylakoid (see outline in **F**), showing lipid bilayers of the thylakoid membranes. **(I)** Carboxysome. **(J)** Close-up showing the protein shell of the carboxysome see in **I**. T, thylakoid; IM, cytoplasmic membrane. White arrows show membranes/shells around ACC inlcuions. Scale bars: **(B**,**C**,**E**,**G**,**H**,**J)** 10 nm; **D,I**: 50 nm.

**FIGURE 9:**
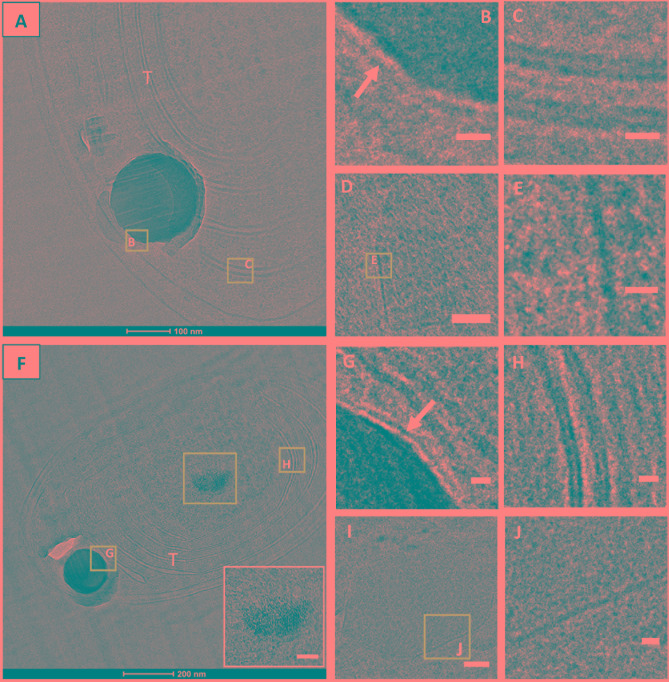
CEMOVIS images. **(A)** *Synechococcus* sp. PCC 6312 cell. **(B)** Close-up of the ACC inclusion, showing a membrane/shell around (arrow). **(C)** Close-up of a thylakoid, showing lipid bilayers. **(D)** Carboxysome. **(E)** Close-up showing the protein shell of the carboxysome seen in **D**). **(F)** *Synechococcus* sp. PCC 6717 cell. The insert shows a close-up of a polyP with a granular texture. **(G)** Close-up of the ACC inclusion outlined in **F**, with a membrane/shell around (arrow). **(H)** Close-up of thylakoids outlined in **F**, showing lipid bilayers. **(I)** Carboxysome. **(J)** Close-up of the protein shell of the carboxysome seen in **I**). T, thylakoid. Scale bars: **(B**,**C**,**E**,**G**,**H**,**J)** 10 nm; **(D**,**I)**: 50 nm.

**FIGURE 10:**
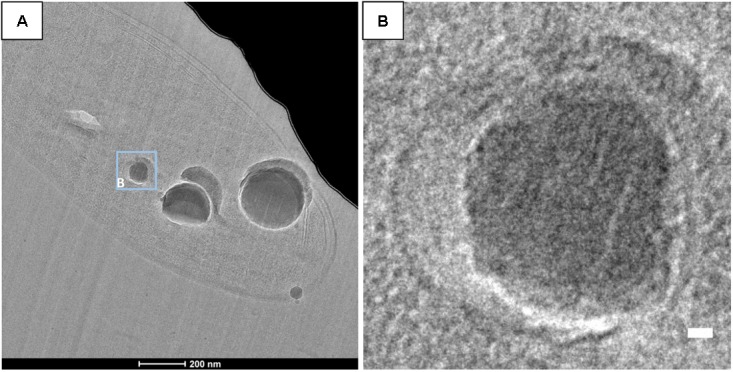
CEMOVIS images of *G. lithophora*. **(A)** Bright field image showing a cell with ACC inclusions. **(B)** Close-up of an ACC inclusion (see location outlined in **A**) enclosed in a wider vesicle. Scale bar: **(B)** 10 nm.

CEMOVIS images taken at a series of defocus values (−1, −2, and −3 μm) confirmed the presence of a thin layer around ACC inclusions (**Figure 7**). The thickness of this layer measured between ∼2.4 and ∼2.7 nm according to the strain (Supplementary Table S3 and **Figures 8**, **9**). This was close to the thickness of the protein shell of carboxysomes, which varied between ∼2.9 and ∼3.5 nm and close to the thickness of the lipid monolayer of a thylakoid membrane (between ∼1.9 and ∼2.2 nm) (Supplementary Table S3 and **Figures 8**, **9**). While the layer was closely circling the ACC inclusions in several cases, the vesicle enclosing the ACC inclusions was sometimes slightly larger with a gap between the inclusion surface and the layer measuring a few tens of nanometers (**Figure 10**).

## Discussion

### Advantages Offered by CEMOVIS for the Study of Intracellular Carbonatogenesis by Cyanobacteria

Li et al. (2016) have been unsuccessful in preserving intracellular ACC inclusions in plastic sections of cyanobacteria. They showed that the use of glutaraldehyde as a fixative agent, followed by OsO_4_ postfixation and dehydration by successive baths of ethanol at room temperature, resulted in complete dissolution of ACC and polyphosphate granules and the precipitation of artefactual Ca- and P-rich particles. This has been suggested as one explanation why intracellular carbonatogenesis by cyanobacteria was overlooked before. ACC are notoriously more labile and soluble than crystalline CaCO_3_ phases (e.g., Brečević and Nielsen, 1989), explaining why they are difficult to preserve. Here, we show that keeping samples frozen throughout fixation and embedding, such as in the freeze-substitution method, is a successful alternative to conventional methods, which preserves ACC inclusions in 500-nm thick sections relatively well with some possible slight chemical modification, including Ca enrichment in polyphosphate bodies. However, ACC inclusions were lost in 70-nm-thick sections. This may be due to the dissolution of ACC when sections float on water after the cut and before retrieval. Alternatively, ACC may be lost mechanically upon sectioning because of the rheological contrast between ACC and resin. Last, uranyl acetate used for staining may also contribute to ACC dissolution and the precipitation of U-rich phases.

The ultrastructures of *Cyanothece sp*. PCC 7425 cells have been thoroughly studied in the past based on plastic ultrathin sections prepared by freeze-substitution (Porta et al., 2000). Consistently with what we observed here, Porta et al. (2000) did not observe ACC inclusions in their ultrathin sections but they detected a high content of electron-transparent granules within the cytoplasm, interpreted as polymers of polyhydroxyalkanoates (PHA). Here, we suggest instead that these granules were dissolved ACC inclusions. Similarly, the ultrastructures and in particular the inclusions within cells of *Synechococcus sp*. PCC 6312 were also recently investigated in details in order to identify the correspondence between ultrastructural features and diverse gene occurrences (Gonzalez-Esquer et al., 2016). The plastic section cut across a cell of *Synechococcus sp*. PCC 6312, as shown in their study, displayed holes close to the pole of the cell between the cytoplasmic membrane and the thylakoids. Based our study, we interpret them as dissolved ACC inclusions.

Our study shows that CEMOVIS well preserved ACC inclusions in sections as thin as 50 nm. Accordingly, several previous studies showed that CEMOVIS offered high preservation quality of bacterial ultrastructures, including lipid bilayers and provided valuable insight into the interactions between minerals and microorganisms (Miot et al., 2011, 2014; Leforestier et al., 2012). In addition to CEMOVIS artifacts such as chatters, compressions, crevasses, and knife-mark damages which have been classically evidenced (Al-Amoudi et al., 2004, 2005; Dubochet et al., 2007; Han et al., 2008; Bleck et al., 2010; Couture-Tosi et al., 2010), we observed here an additional artifact more specific to intracellularly calcifying cyanobacteria and affecting ACC, consisting in a smear of carbonate leaking out of the inclusions. Moreover, we observed varying responses to fracturing among ACC inclusions: while some showed fractures perpendicular to the cut direction, suggesting some brittleness, other were very smooth. Different chemical types of ACC phases have been evidenced by previous studies based on their hydration level: type 1 which is hydrated ACC and type 2 which corresponds to anhydrous ACC (e.g., Gong et al., 2012). Whether the difference of brittleness observed between ACC inclusions within cyanobacterial cells may correspond to different degrees of ACC dehydration remains to be further investigated.

### Precipitation of Amorphous Calcium Carbonate Occurs in a Compartment

Although, lipid vesicles have been suggested to enclose crystalline calcite microspheres in the giant sulfur oxidizing bacterium *A. oxaliferum* based on TEM observations of sections prepared by conventional ultramicrotomy (De Boer et al., 1971; Head et al., 2000; Gray and Head, 2014), vesicles enclosing mineral phases are notoriously difficult to observe by TEM as emphasized by studies of magnetotactic bacteria (e.g., Komeili et al., 2006). Indeed, it has been noticed that a membrane tightly bound to a mineral is difficult to visualize due to the adverse observation of Fresnel fringes in out-of-focus images or the possibly overwhelming diffraction contrast of mineral phases. Cryo-electron tomography of cells preserved in their native state in vitreous ice has been used to unambiguously evidence lipid vesicles enclosing magnetites in diverse strains of magnetotactic bacteria (e.g., Komeili et al., 2006; Abreu et al., 2013). Here, CEMOVIS images taken at different defocus unambiguously showed the presence of a ∼2.5-nm-thick layer enveloping ACC inclusions in all cyanobacteria forming intracellular carbonates. CEMOVIS did not offer additional clue on the exact nature of this layer. However, based on its thickness and its appearance as a single electron dense line, it can be concluded that this layer is not a lipid bilayer. In that sense, intracellular calcification by cyanobacteria seems to differ from the above mentioned cases of intracellular biomineralization in magnetotactic bacteria and *A. oxaliferum*. Instead, based on the thickness of this layer surrounding ACC inclusions, it can be speculated that it is either (1) a lipid monolayer or (2) a protein shell, such as the one enclosing carboxysomes.

Phospholipid monolayers, appearing as single electron-dense lines by CEMOVIS and measuring ∼2.5 nm in width have been observed repeatedly around lipid droplets in eukaryote cells and PHA inclusions in prokaryote cells (Tauchi-Sato et al., 2002; Wältermann and Steinbüchel, 2005). Cryo-TEM, and CEMOVIS specifically, have been noted to be particularly efficient in revealing such monolayers (Bleck et al., 2010). It has been suggested that their hydrophilic headgroup face the cytoplasm, while the hydrophobic acyl chains extend into the lipid droplet content (Brown, 2001). Interestingly, it has been shown that confinement in micelles composed of amphiphilic molecules such as phospholipids stabilizes ACC particles by excluding water and therefore preventing reorganization of ACC into a crystalline phase (e.g., Tester et al., 2011; Liu et al., 2012). Moreover, many studies have synthesized CaCO_3_ phases in the presence of amphiphilic molecules, including phospholipids (e.g., Pal et al., 2010). However, in these studies, precipitation always occurred on the hydrophilic head group side in accordance with the hydrophilicity property of CaCO_3_ nanoparticles. Overall, while this hypothesis of a lipid monolayer surrounding ACC inclusions in cyanobacteria has some interesting implications, it faces two major issues: (1) we have not evidenced any lipid droplet in the cells of studied species and (2) hydrophilic headgroups are expected to face the cytoplasm, whereas ACC granules appear on the other side, i.e., within the vesicles.

An alternative hypothesis is that the layer around ACC inclusions is a protein shell. A large diversity of microcompartments, enclosed by protein shells has been described in diverse bacteria, including cyanobacteria (e.g., Kerfeld et al., 2010). Well-known examples of microcompartments in cyanobacteria are carboxysomes, which enclose RubisCO and carbonic anhydrases within a 3 to 4-nm thick proteinaceous shell (e.g., Cannon et al., 2001). Several other types of microcompartments have been described in bacteria as well, such as Pdu or Eut, involved in 1,2-propanediol and ethanolamine utilization, respectively, and often referred to as polyhedral bodies because of their resemblance with carboxysomes in size, shape and electron density (Kerfeld et al., 2010). Gas vesicles, appearing as spindle-shaped or cylindrical electron-transparent structures and surrounded by a ∼2 nm thick wall composed of a single layer of proteins are additional bacterial microcompartments, that can be found in some cyanobacteria (Pfeifer, 2012). Li et al. (2016) hypothesized that carboxysomes may act as nucleation sites for ACC precipitation within cyanobacterial cells. Indeed, CO_2_ is fixed within carboxysome and therefore a higher activity of CO_3_^2−^ may be expected at this location. Here, we did not observe ACC inclusions in ascertained icosahedral compartments. However, the canonical icosahedral shape of carboxysomes has been shown to sometimes become irregular or elongated in bacteria (Iancu et al., 2010). Moreover, it has been suggested that pH variations and interactions with metallic cations may modify the shape of such compartments from spherical to faceted shapes (Olvera de la Cruz, 2016). Finally, some proteins have been shown to stabilize ACC (Rao et al., 2013) but this has never been tested on proteins composing the shell of bacterial microcompartments.

## Conclusion

CEMOVIS offered unique advantages to image the ultrastructures of cyanobacterial cells forming intracellular carbonates. Both freeze-substitution and CEMOVIS evidenced the presence of a thin layer around ACC inclusions in cyanobacteria. The nature of this layer, a lipid monolayer or a protein shell, remains enigmatic, but in any case, this intracellular compartment involved in biomineralization adds to the numerous intracellular microcompartments already known in cyanobacteria. Such a compartment isolates the site where ACC precipitates from the periplasm, and possibly allows the achievement of a high pH and/or [Ca^2+^] as needed for ACC precipitation, while the cytoplasm remains at a neutral pH and low [Ca^2+^] as speculated by Cam et al. (2018). The creation of special chemical conditions allowing ACC precipitation within this compartment may involve Ca^2+^ and/or H^+^ transport. However, the molecular actors involved in the functioning of this compartment have yet to be discovered.

## Author Contributions

MB, KB, and MS were responsible for the design of the study. MB, MS, CB, CG, J-MG, FS-P, MP, CF, and JM performed the experiments. MB, MS, CB, and KB interpreted the findings and drafted the manuscript. All authors critically reviewed content and approved the final version for publication.

## Conflict of Interest Statement

The authors declare that the research was conducted in the absence of any commercial or financial relationships that could be construed as a potential conflict of interest.
