# Supplementary material for: Amorphous Calcium Carbonate Granules Form Within an Intracellular Compartment in Calcifying Cyanobacteria
Source: Front Microbiol. 2018 Aug 6;9:1768. doi: 10.3389/fmicb.2018.01768 (PMC6087745; doi:10.3389/fmicb.2018.01768)
Supplement: Supplementary file 1 [file Data_Sheet_1.PDF]

Supplementary Material

Amorphous calcium carbonate granules form within an intracellular compartment in calcifying cyanobacteria

Marine Blondeau, Martin Sachse, Claire Boulogne, Cynthia Gillet, Jean-Michel Guigner, Fériel Skouri-Panet, Mélanie Poinsot, Céline Ferard, Jennyfer Miot, Karim Benzerara*

*** Correspondence:** Corresponding Author: karim.benzerara@upmc.fr

1 Supplementary Figures and Tables

1.1 Supplementary Figures

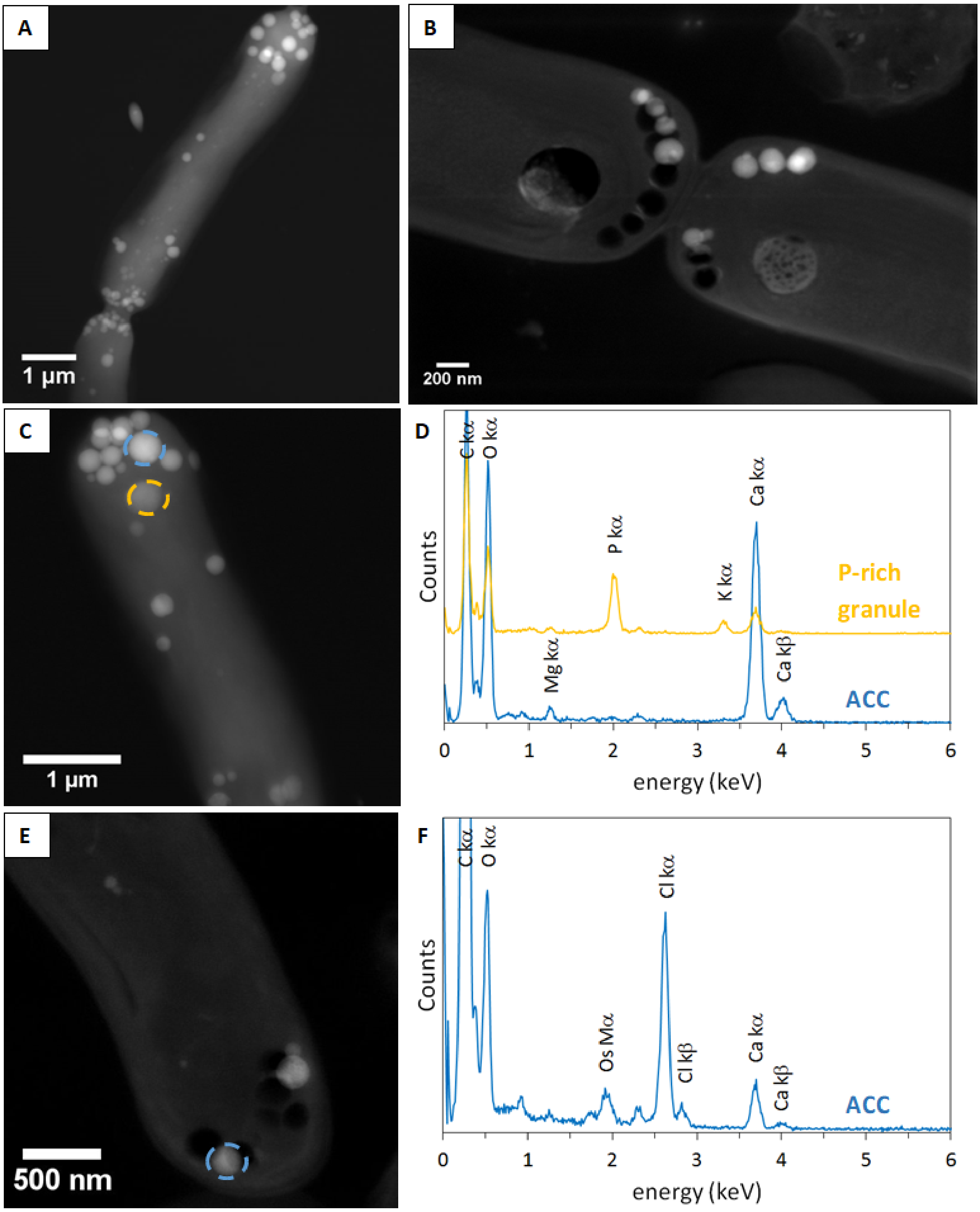

Supplementary Figure 1. STEM analyses of *Thermosynechococcus* sp. BP-1. (**A**) STEM-HAADF image of whole cells. (**B**) 350-nm-thick unstained section. P: P-rich granule. (C) STEM-HAADF image of whole cells. (**D**) EDXS spectra one of an ACC inclusion (blue) and a P-rich granule (yellow), see **C** for location. (E) 500-nm-thick unstained section of a cell. (**F**) EDXS spectrum of an ACC inclusion, see **E** for location.

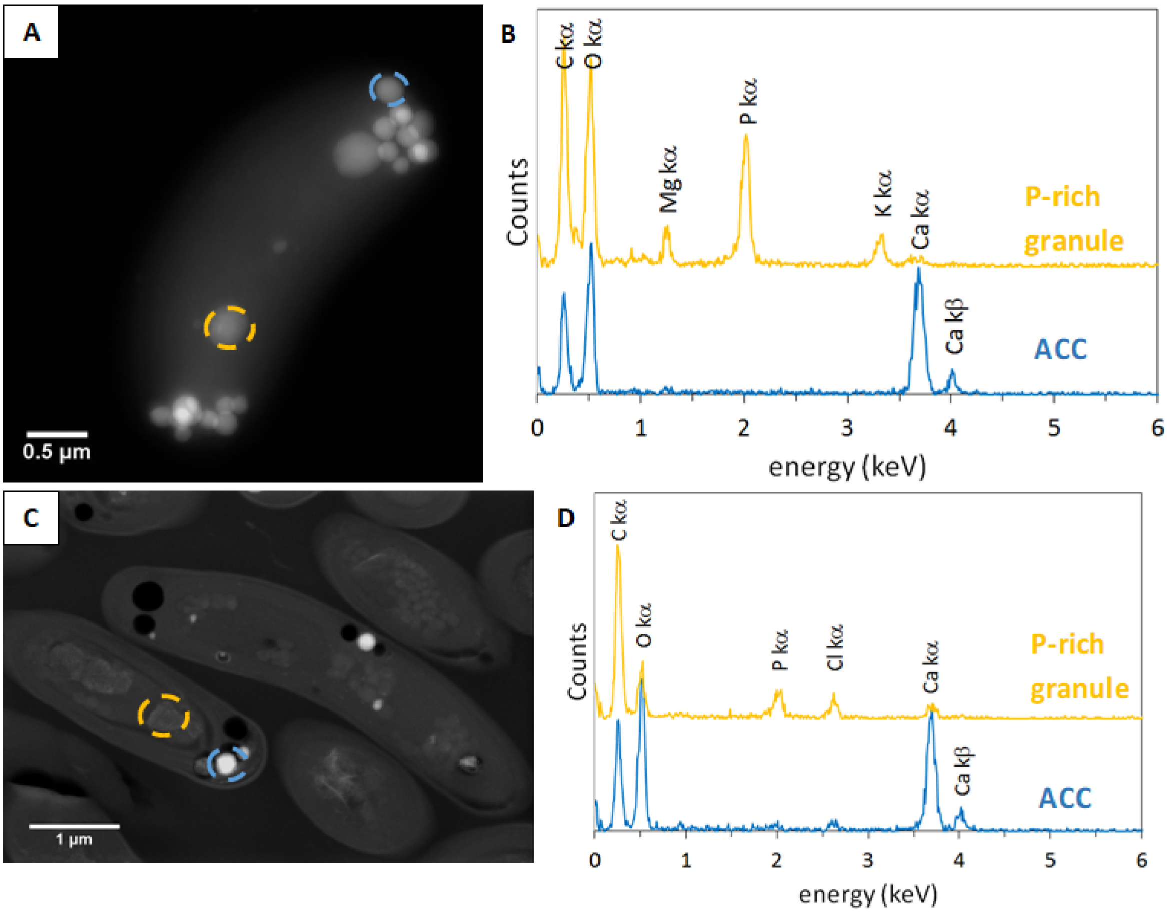

Supplementary Figure 2: STEM analyses of *Synechococcus calcipolaris* cells. (**A**) STEM-HAADF images of an un-sectioned air-dried cell. (**B**) EDXS spectra of an ACC inclusion (blue) and a P-rich granule (yellow), see **A** for location. (**C**) 250-nm-thick unstained section of cells. (**D**) EDXS spectra of an ACC inclusion (blue) and a P-rich granule (yellow), see **C** for location.

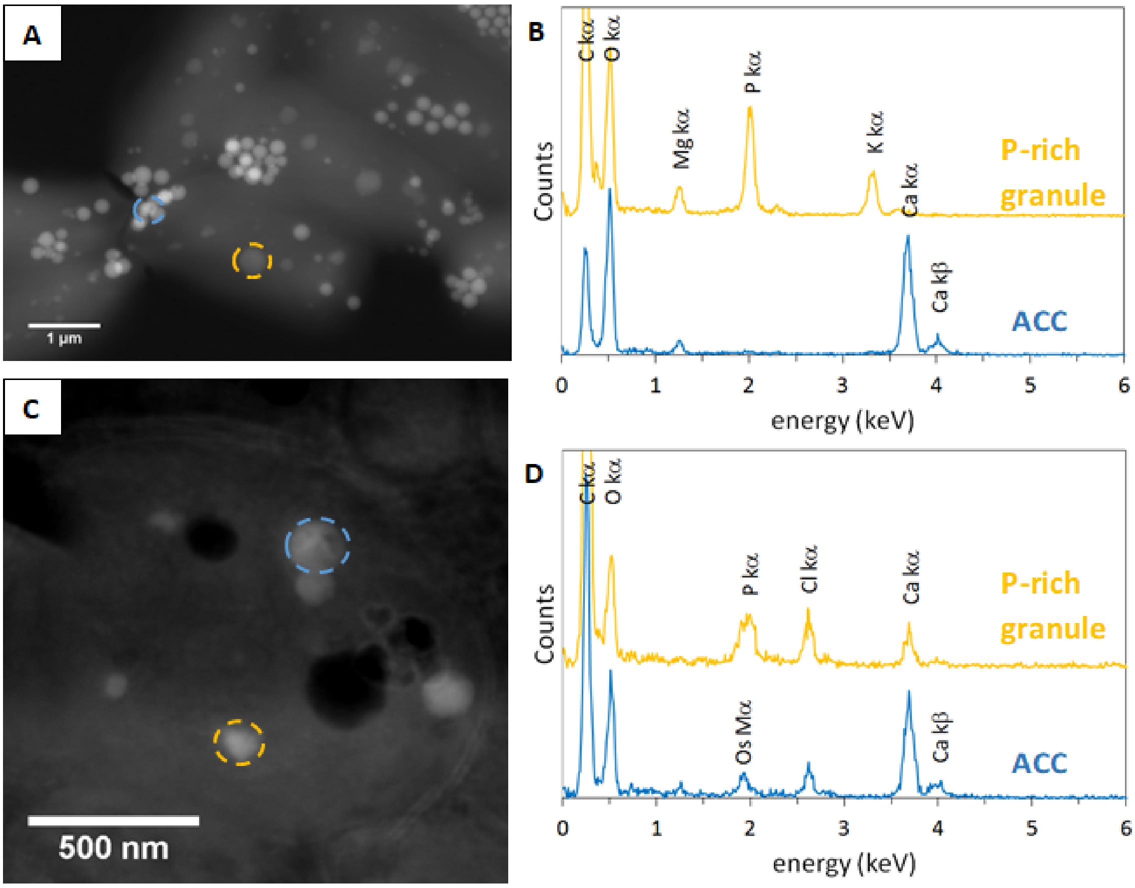

Supplementary Figure 3. STEM analyses of *Synechococcus* sp. PCC 6312. (**A**) STEM-HAADF images of un-sectioned air-dried cells. (**B**) EDXS spectra of an ACC inclusion (blue) and a P-rich granule (yellow), see **A** for location. (**C**) 500-nm-thick unstained section of one *Synechococcus* sp. PCC 6312 cell. (**D**) EDXS spectra of an ACC inclusion (blue) and a P-rich granule (yellow), see **C** for location.

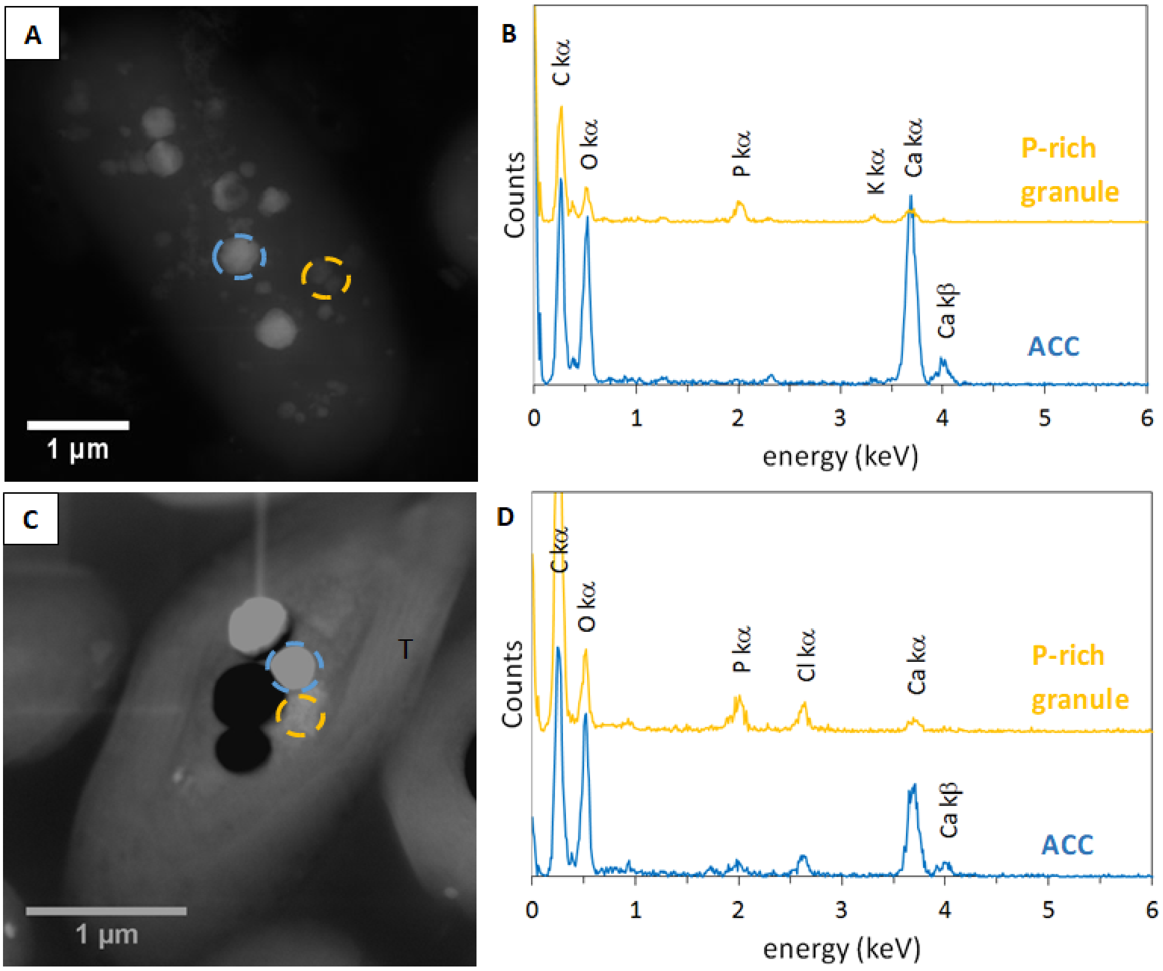

Supplementary Figure 4. STEM analyses of *Cyanothece* sp. PCC 7425. (**A**) STEM-HAADF image of an un-sectioned air-dried cell. (**B**) EDXS spectra of an ACC inclusion (blue) and a P-rich granule (yellow), see **A** for location. (**C**) 500-nm-thick unstained section of a cell. (**D**) EDXS spectra of an ACC inclusion (blue) and a P-rich granule (yellow), see **C** for location. T: thylakoid.

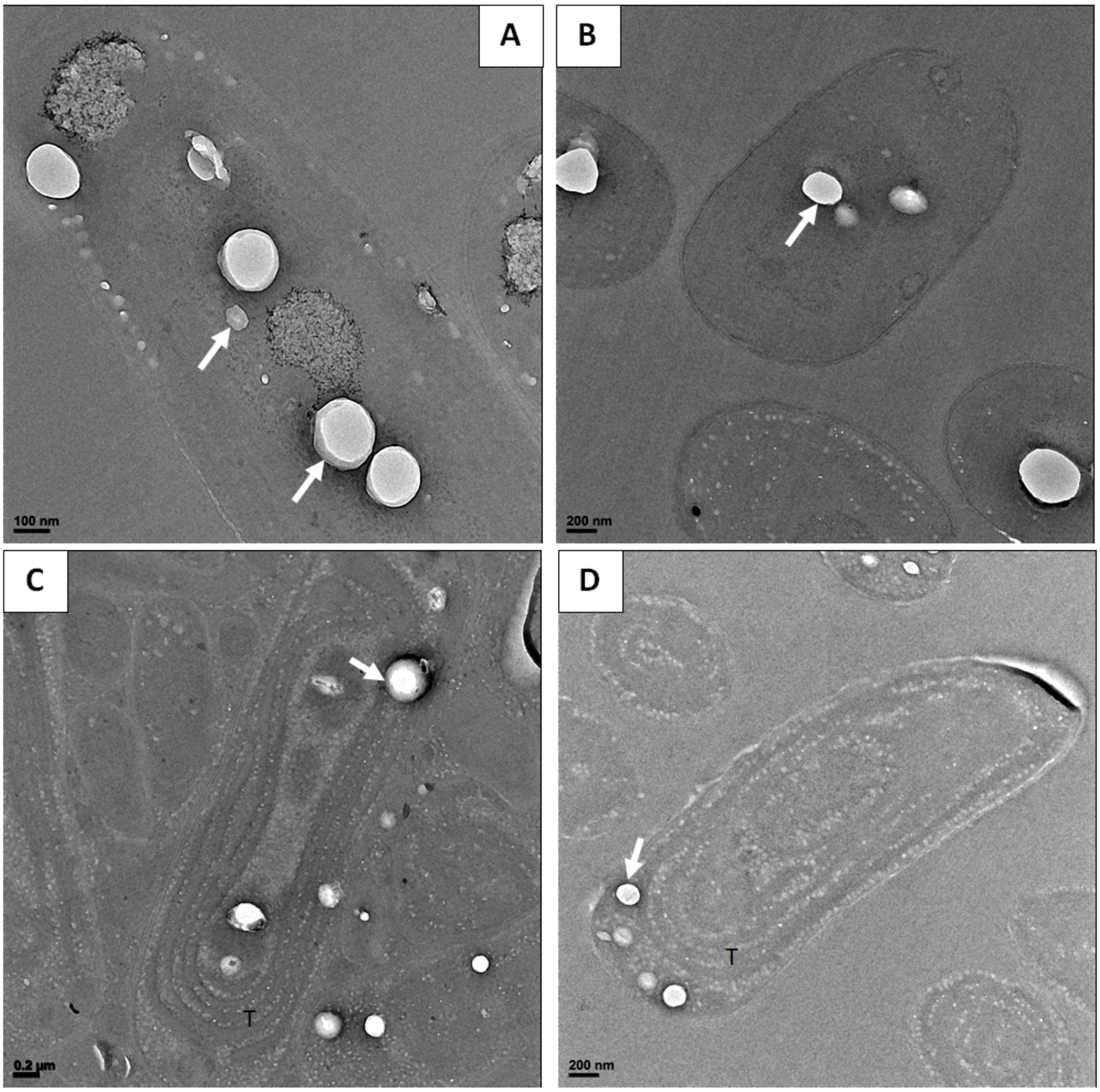

Supplementary Figure 5: TEM images of unstained 70-nm-thick sections. (**A**) *Gloeomargarita lithophora*. (**B**) *Cyanothece* sp. PCC 7425. (**C**) *Synechococcus* sp. PCC 6312. (**D**) *Synechococcus* sp

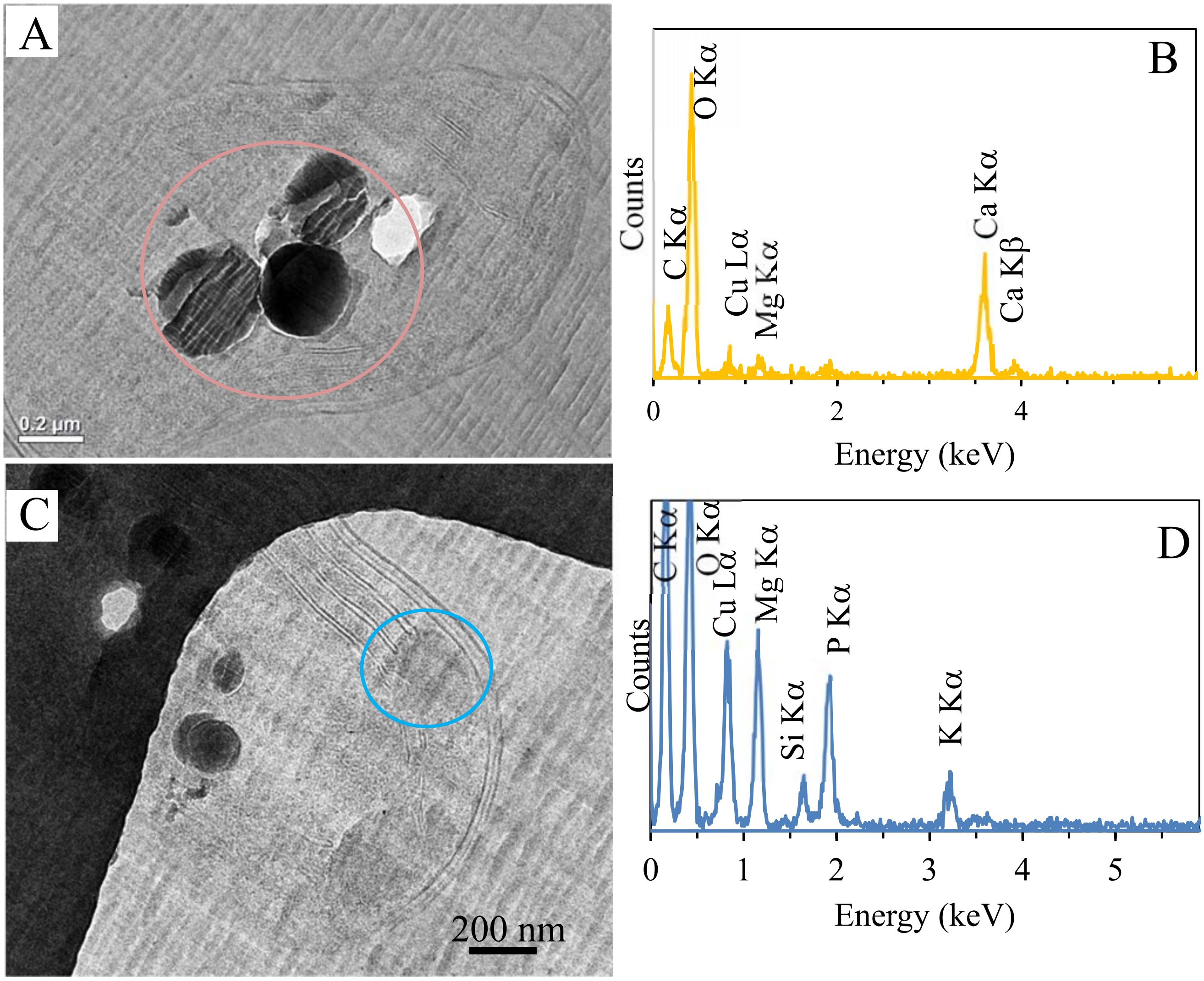

Supplementary Figure 6. EDXS analyses of CEMOVIS sections. (**A)** CEMOVIS image of a *G. lithophora* cell. (**B**) EDXS spectrum of ACC inclusions outlined by the orange circle in (**A**). **(C)** CEMOVIS image of a *G. lithophora* cell. (**D)** EDXS spectrum of the P-rich granule outlined by in (**C**).

1.2 Supplementary Tables

	Freeze-substitution		CEMOVIS	
**Strains**	**OD 730 nm**	**Cell/mL**	**OD 730 nm**	**Cell/mL**
G. lithophora C7	0.638	5.72*10^7	1.065	9.54*10^7
Cyanothece sp. PCC 7425	1	2.98*10^7	0.976	2.91*10^7
Synechococcus sp. PCC 6717	1.760	6.65*10^7	0.523	1.98*10^7
Synechococcus sp. PCC 6312	1.400	7.42*10^7	1.93	10.2*10^7
Synechococcus calcipolaris G9	1.512	9.69*10^7	-	-
Synechococcus elongatus BP-1	1.428	3.64*10^7	-	-

Supplementary Table 1: Optical density values at 730 nm and corresponding cell densities (cell/mL) of the cultures used for freeze-substitution and CEMOVIS.

	**Carboxysomes**		
**Strains**	**Length (nm)**	**Width (nm)**	**Number of measured carboxysomes**
G. lithophora	130 +/- 26	76 +/- 16	n=4
Cyanothece sp. PCC 7425	230 +/- 62	153 +/- 40	n=5
Synechococcus sp. PCC 6717	241 +/- 63	192 +/- 24	n=4
Synechococcus sp. PCC 6312	267 +/- 46	182 +/- 51	n=3

Supplementary Table 2. Carboxysome length and width (in nanometers) measured by CEMOVIS for *G. lithophora*, *Cyanothece* sp. PCC 7425, *Synechococcus* sp. PCC 6717, and *Synechococcus* sp. PCC 6312.

Strains	Thickness membrane/shell around ACC (nm)	Thickness thylakoid membrane (nm)	Thickness cytoplasmic membrane (nm)	Thickness carboxysome shell (nm)
G. lithophora C7	2.7 +/- 0.5	5.2 +/- 0.5	5.6 +/- 0.7	2.9 +/- 0.4
Cyanothece sp. PCC 7425	2.4 +/- 0.4	5.2 +/- 0.9	5.5 +/- 0.6	3.5 +/- 0.5
Synechococcus sp. PCC 6717	2.4 +/- 0.7	6.1 +/- 0.8	6.8 +/- 0.5	3.3 +/- 0.6
Synechococcus sp. PCC 6312	2.5 +/- 0.4	5.6 +/- 0.8	6.1 +/- 0.8	3.5 +/- 0.8

Supplementary Table 3. Thickness in nanometers of the membrane/shell around ACC inclusions, the membrane of thylakoids, the cytoplasmic membrane, and the protein shell of carboxysomes, as determined by CEMOVIS.
